# A mini-review: Advances in plant-derived extracellular vesicles as nano-delivery systems for tumour therapy

**DOI:** 10.3389/fbioe.2022.1076348

**Published:** 2022-12-15

**Authors:** Ying Zhu, Xiaona Zhou, Zheng Yao

**Affiliations:** ^1^ Department of Basic Medical, Yunnan University of Chinese Medicine, Kunming, China; ^2^ Department of First Clinical Medical, Yunnan University of Chinese Medicine, Kunming, China; ^3^ Key Laboratory of Microcosmic Syndrome Differentiation, Education Department of Yunnan, Kunming, China

**Keywords:** extracellular vesicles, plant-derived extracellular vesicles, nano-delivery system, tumour therapy, brain tumours, breast cancer

## Abstract

Extracellular vesicles are functionally active, nanoscale, membrane-bound vesicles that can be secreted by all cells. They have a key role in most health and disease states and have gradually become a promising class of delivery vehicles for targeted therapies for a variety of diseases. Plant-derived extracellular vesicles have received increasing attention based on their easy availability, non-toxicity and high absorption. However, compared with mammalian extracellular vesicles, the role of these nanoparticles as nano-delivery systems in tumour therapy has been underestimated. In this paper, the application of plant-derived extracellular vesicles and their nano-derivatives as nano-delivery systems in tumour therapy is reviewed to illustrate their great application potential.

## 1 Introduction

Cancer has become the largest global health problem because of the high recurrence and mortality rates (Sung et al., 2021). Effective treatment solutions are severely restrained because of late diagnosis, drug resistance, metastasis and poor prognosis (Zhang et al., 2022). Currently, chemotherapy is one of the main treatments for cancer and has made considerable therapeutic progress and prolonged the life of tens of millions of people (Huyan et al., 2020). However, the low delivery rate of therapeutic drugs to tumour cells and substantial toxic side effects on healthy cells seriously restrict the curative effect of chemotherapy (Senapati et al., 2018). Therefore, an efficient and safe drug delivery system is urgently needed to reduce the side effects of chemotherapy and improve the prognosis of patients.

The first discovery of plant-derived extracellular vesicles (PDEVs) was attributed to multivesicular bodies discovered by Jensen from cotton in 1965 (Jensen, 1965). Subsequent studies demonstrated that PDEVs contribute to the development of early defence structures to counteract pathogens. However, their biological function remained obscure for a long time (Urzì et al., 2022). With continued research, the understanding of PDEVs has become more comprehensive, especially in recent years and there is now a more confidant understanding of their physiological characteristics. The structural characteristics of PDEVs are similar to exosomes in extracellular vesicles in animals (Wang et al., 2022). PDEVs are membrane structures with lipid bilayers, surface membrane and internal proteins, nucleic acids and other substances (Zhang et al., 2016a). The particle size is usually slightly larger than that of animal exosomes (Man et al.). PDEVs are produced by a variety of plants, can be isolated from a range of edible vegetables and fruits and are involved in various physiological processes and intercellular communications (Yu et al.,; Urzì et al., 2022; Zhang et al., 2022).

PDEVs are considered potential alternatives to synthetic liposomes or nanoparticles. Therefore, vesicles of plant origin have the potential to develop green, sustainable and biocompatible materials to deliver bioactive compounds (Srivastava et al., 2022). In this mini-review, we summarise the current advantages of PDEVs as a nano-delivery system and research progress in tumour therapy to highlight their use as a more advantageous chemotherapy drug delivery system.

## 2 Advantages of plant-derived extracellular vesicles as nano-delivery systems

### 2.1 Safety

Compared with the cancer-stimulating risk of animal extracellular vesicles (Tan et al., 2021) and the toxicity of synthetic lipid carriers (Palazzolo et al., 2018), PDEVs are very safe. Because they are natural nanoparticles secreted by plants and already contained in foods currently consumed by humans, they are tolerated by the immune system and have good biocompatibility (Anusha and Priya, 2022). Both animal and cellular experiments have shown them to be non-toxic (Wang Q. et al., 2013; Raimondo et al., 2015; Zhuang et al., 2015; Umezu et al., 2021), and even PDEVS encapsulation can reduce the side effects of DOX to improve efficacy (Zhang et al., 2016c). They do not pass through the placental barrier (Wang Q. et al., 2013) and they show favourable safety profiles in organ pathology and haemolytic reaction as well as levels of cellular inflammatory factors (Dad et al., 2021; Sarvarian et al., 2022).

### 2.2 Low cost

Plant-derived extracellular vesicles are mostly derived from common plants, with a common source of raw materials that can be produced economically on a large scale. Compared with animal cell-derived EVs, PDEVs not only reduce the cost of cell culture supply but also reduce the time and labour of large scale cell culture. PDEVs reduce the cost/yield ratio of animal cell-derived EVs by approximately 300-fold (Li et al., 2018). This facilitates increased productivity and can be used on a large scale in the clinic.

### 2.3 High stability

The lipid membranes of PDEVs protect bioactive substances from external substances, including changes in pH value and heat and light and have extremely high stability (Logozzi et al., 2021). PDEVs are kept in circulation in the body for a long time, which is conducive to the continuous accumulation of drug effects (Yu et al.,; Wang Q. et al., 2013). At the same time, PDEVs are tolerated and remain stable in the gastrointestinal environment. Grape-derived EVS were resistant to degradation of saliva, gastric acid, and proteolytic enzymes and were able to spread through the intestinal tract, migrate through intestinal mucus and eventually be absorbed by mouse intestinal stem cells (Ju et al., 2013). Ginger-derived EVS were highly stable in simulated gastrointestinal fluids (Zhang et al., 2016a). In a study by Wang et al. grapefruit-derived EVS remained stable in both acid-based and simulated gastrointestinal solutions, exhibiting excellent anti-digestive capacity (Wang et al., 2014).

### 2.4 Biological activity

PDEVs, as natural vectors, inherently carry the innate active compounds of the source plant. This makes PDEVs have good biological functions such as antioxidant, anti-inflammatory and anti-cancer. It has been shown that EVs derived from lemon carry micronutrients such as vitamin C and citrate, which exert antioxidant effects in human cells (Akuma et al., 2019). Broccoli-derived EVs, containing sulforaphane, an active component found in some vegetables, contributed in the prevention of colitis in mice (Deng et al., 2017). Ginger-derived nanoparticles were found to carry 6-gingerol and 6-shogaol, two anticancer, anti-inflammatory and antioxidant bioactive compounds, and good hepatoprotective effects (Zhuang et al., 2015; Zhang et al., 2016b). Thus, PDEVs as nano-delivery systems can synergize with loaded drugs to exert therapeutic potential to improve efficacy.

Therefore, based on the above advantages, PDEVs have significant potential in tumour treatment.

## 3 Application of plant-derived extracellular vesicles as the nano-delivery system in tumours

### 3.1 Application in brain tumours

The main obstacle to the development of drugs in the central nervous system is the lack of access to the brain. A large number of drugs with the potential to treat brain tumours are not widely used because they cannot cross the blood–brain barrier depending on the therapeutic concentration (Zhuang et al., 2016). Wang et al. recombined lipids derived from grapefruit to form a grapefruit-derived nanovector (GNV). *In vivo* biological distribution of DiR-labelled GNVs assessed in mouse studies after intranasal administration found that most of these continued to be stably distributed in the brain; furthermore, *in vitro* experiments showed that GNVs can encapsulate functional siRNAs and efficiently deliver them to mouse glioma GL26 cells. This study also found that intranasal administration of GNVs that encapsulated with the Stat3 inhibitor JSI-124 significantly inhibited Stat3 activation in a mouse model of brain tumours planted with GL26 cells. Delays in tumour growth thereby increased the survival rate of brain tumour mice (Wang Q. et al., 2013).

In further studies, Zhuang et al. (Zhuang et al., 2016) demonstrated that GNVs can carry microRNA (miR)-17 to treat brain tumours in mice. This study shows that the targeting of GNV coated with folic acid (FA) to cells is enhanced and that FA-GNVs can bind to folic acid receptors GL-26 brain tumour cells. In addition, FA-GNV-coated polyethyleneimines (FA-pGNVs) not only enhance the ability to carry RNA but also the toxicity of polyethyleneimines is eliminated by using GNVs. Intranasal administration of brain tumour mice with FA-pGNV/miR-17 can quickly deliver miR-17 to mouse brains and inhibit expression of major histocompatibility complex I (MHCI) on GL-26 cells to trigger activation of natural killer (NK) cells thereby killing tumour cells and playing a role in treating brain tumours in mice. The above studies reported that PDEVs can be used as nanocarriers to help therapeutic drugs enter the brain through the blood–brain barrier, and in combination with other modifiers, can effectively and stably fulfil the role of drugs in treating brain tumours.

### 3.2 Application in colon cancer

Colon cancer is the third most common cancer globally, and recently, the incidence of colon cancer has grown year by year, increasing what is already a threat to human life and health (Sung et al., 2005; Twelves et al., 2005; Yeo et al., 2015; Arnold et al., 2017). Chemotherapy remains the most common treatment option for colon cancer patients (de Gramont et al., 2012). However, toxic sie effects of chemotherapeutic drugs constrain their efficacy. Therefore, a nano-delivery system that can both maintain (or improve) the effect of drug treatment and reduce associated toxicity has broad prospects. Zhang et al. (Zhang et al., 2016c) reported an advance in treating colon cancer using a ginger-derived nano-GDLV modified with FA modifications of the therapeutic agent doxorubicin (DOX) to successfully inhibit tumour growth in colon-26 xenograft tumour models. This study shows that FA-GDLV has an increased ability to target colon-26 tumours compared to unmodified GDLV, possibly through active FA-FUR interactions. However, the accumulation of FA-GDLV in the spleen and liver was found to be significantly less than that with GDLV, suggesting that using FA-GDLV as a carrier can reduce the systemic toxicity of the drug to normal tissues. *In vivo* studies reported that FA-GDLV is detectable after 48 h of circulation after intravenous injection, giving them a greater chance of penetrating the tumour. Therefore, FA-GDLV can be an ideal drug delivery platform that can exert anti-tumour effects while avoiding adverse reactions of free-circulating drugs.

Li et al. (Li et al., 2022) performed *in vitro* anticancer studies using broccoli-derived extracellular vesicle-coated astaxanthin nanoparticles that had a more significant inhibitory effect on the proliferation of human colon cancer HT-29 cells than that of astaxanthin alone. Other studies reported that GNVs, after modification, can effectively deliver chemotherapy drugs to colon cancer cells and significantly inhibit tumour growth in colon cancer model mice (Wang Q. et al., 2013; Wang et al., 2015).

PDEVs have been introduced into clinical trials. Researchers from the James Graham Brown Cancer Centre used plant-derived nano-vesicle-coated curcumin to treat intestines in cancer patients. Curcumin has a powerful inhibitory effect on colon cancer cell lines; however, because of the low solubility, poor stability and simple metabolism, oral curcumin exhibits only limited bioavailability even at very high doses. By using PDEV delivery, most of the major obstacles to curcumin application can be addressed, including increased solubility, bioavailability and stability. This remains ongoing work, so further clarification of the conclusions is needed (NCT01294072).

### 3.3 Application in breast tumours

Breast cancer as a malignancy seriously threatens the health of women globally (Yu et al., 2021). In particular, once metastasis occurs in the later stages of breast cancer, a poor prognosis can mean limited life expectancy for the patient (Chaffer and Weinberg, 2011). Currently, there are still several defects in commonly used chemotherapy and photothermal therapy, such as tumour cell resistance, insufficient accumulation of drug properties and non-specific distribution and toxicity to normal organs, which seriously limit their application (Minko et al., 2013; Liu et al., 2019; Chen et al., 2021).

Zeng et al. (Zeng et al., 2022) argue that to overcome the drawbacks of traditional treatment methods, a functional nanocarrier loaded with mixed anticancer drugs has favourable prospects in breast cancer treatment. They found that aloe vera gel-derived nanovesicles (gADNV) is an example of an excellent nanocarrier in this regard. gADNV is modified with an active integrin-targeted peptide (Arg–Gly–Asp, RGD) to effectively deliver the photothermal drug indocyanine green (ICG) and DOX for breast cancer treatment. The ‘competitive’ relationship between ICG and DOX can be transformed into a ‘cooperative’ relationship by *π*–*π* stacking interactions within gADNVs to improve their loading efficiency. This dual-drug co-delivery nanosystem called DIARs has good stability, leak resistance and exhibits high breast tumour targeting capabilities both *in vitro* and *in vivo*. Simultaneously, this nanosystem significantly inhibits cell growth and migration and induces apoptosis through a combination of phototherapy and chemotherapy. Intravenous administration of DIARs showed high therapeutic efficacy in 4T1 tumour-bearing mouse models and no significant damage to other organs. Wang et al. (Wang et al., 2015) confirmed that the DOX and curcumin can be successfully delivered to the desired site of inflammation through GNVs and achieved therapeutic effects by modifying GNVs with individually activated leukocyte membranes (IGNV). *In vivo* experiments also proved that intravenous injection of IGNV-DOX or IGNV-Cur can significantly inhibit the growth of breast tumours in mice. These studies showed that PDEVs can be successfully combined with various modifications to improve the targeting and loading efficiency of breast cancer treatment drugs and reduce their side effects to give full play to their therapeutic roles, providing an effective approach in treating breast tumours.

### 3.4 Application in oral cancer

Oral squamous cell carcinoma (OSCC) is the most common oral malignancy characterised by rapid local invasion and a high recurrence rate (Chi et al., 2015). Patients with recurrent OSCC have been reported to have failed treatment due to resistance to chemotherapy drugs, resulting in low survival rates (Wang B. et al., 2013; Wang et al., 2016). 5-Fluorouracil (5-FU) is one of the most used chemotherapy drugs for treating OSCC, although the anti-tumour efficacy is affected by side effects and tumour resistance (Longley et al., 2003; Atashi et al., 2021). Meng et al. (Yang et al., 2021) found that bitter melon-derived extracellular vesicles (BMEVs) are an intrinsically biologically active form of cancer treatment and potential nano-delivery carriers. BMEVs combined with 5-FU can enhance OSCC apoptosis by increasing the production of reactive oxygen species while BMEVs can enhance the cytotoxic effect of 5-FU during OSCC treatment and reduce resistance by downregulating the expression of NLRP3.

Another study reports that ginger-derived exosome-like nanovesicles (GDENs) can be engineered to inhibit tumours *via* intravenous delivery of siRNA by exhibiting ligands. The researchers mixed GDENs with arrow tail RNA nanoparticles and modified these with FA and a ligand of arrow tail RNA to prepare a GDEN carrying siRNA that targets the BIRC5 gene. *In vitro* experiments demonstrated that GDEN modified to carry siRNA can effectively bind to human oral epithelial cancer kB cells and effectively knockdown target gene expression. The results of *in vivo* experiments showed that mice with oral epithelial cell carcinoma xenotransplanted from humans could be injected intravenously with GDEN loaded with siRNA, which can significantly inhibit tumour growth in mice (Li et al., 2018). This, PDEVs have the potential to treat nano transmission systems for oral cancer.

### 3.5 Application in melanoma

Zeng et al. identified a nanocarrier made of aloe vera that is stable and leak-proof. Nanovesicles were isolated from the gel and outer skin of aloe vera (gADNVs and rADNVs) with high quality and yield. gADNVs exhibits good structural and storage stability, oxidation resistance and impermeability. They can be efficiently absorbed by melanoma cells and are not toxic *in vitro* or *in vivo*. Indocyanine green (ICG) loaded in gADNVs (ICG/gADNVs) showed high stability in both a heated system and in serum, and the retention rate exceeded 90% after 30°days stored in gADNVs. This high degree of retention for ICG/gADNVs can enable them effectively destroy melanoma cells and inhibit the growth of melanoma and is superior to treatment using free ICG and ICG liposomes. Interestingly, ADNVs exhibited significant penetration of mouse skin, which may facilitate non-invasive transdermal administration (Zeng et al., 2021).

### 3.6 Application in liver metastasis of colon cancer

Liver metastases are the cause of cancer death in most patients (de Ridder et al., 2016; Milette et al., 2017). Effective treatment of metastatic liver tumours is not currently available (Alberts and Wagman, 2008; Sabanathan et al., 2016). Teng et al. (Teng et al., 2016) found that miR-18a wrapped in GNVs mediates inhibition of liver metastasis, depending on the induction of M1 (F4/80 + IFNγ + IL-12+) macrophages. The depletion of macrophages eliminates the anti-metastatic effect. In addition, IFNγ induction of macrophages mediated by targeting IRF2-mediated miR-18a is required for the subsequent induction of IL-12. IL-12 then activates NK and NKT cells to inhibit liver metastasis in colon cancer. Knockout of IFNγ expression eliminates miR-18a-mediated IL-12 induction, and miR-18a therapy has an anti-metastatic effect in T cell-deficient mice, but no anti-metastatic effect on NK and NKT-deficient mice. Co-delivery of miR-18a and siRNA IL-12 to macrophages does not lead to activation of co-cultured NK and NKT cells.

## 4 Discussion

At present, the research on drug delivery based on PDEVs is still in its infancy, which is different from the research on animal extracellular vesicles that has become increasingly mature. However, the advantages of PDEVs as a natural nanovector cannot be ignored. This mini-review summarises the current role of PDEVs as a nano-delivery system in tumour treatment in detail and highlights the significant potential in the treatment of tumours. This review provides several ideas and directions for the study of tumour treatment protocols and nanovectors.

**FIGURE 1:**
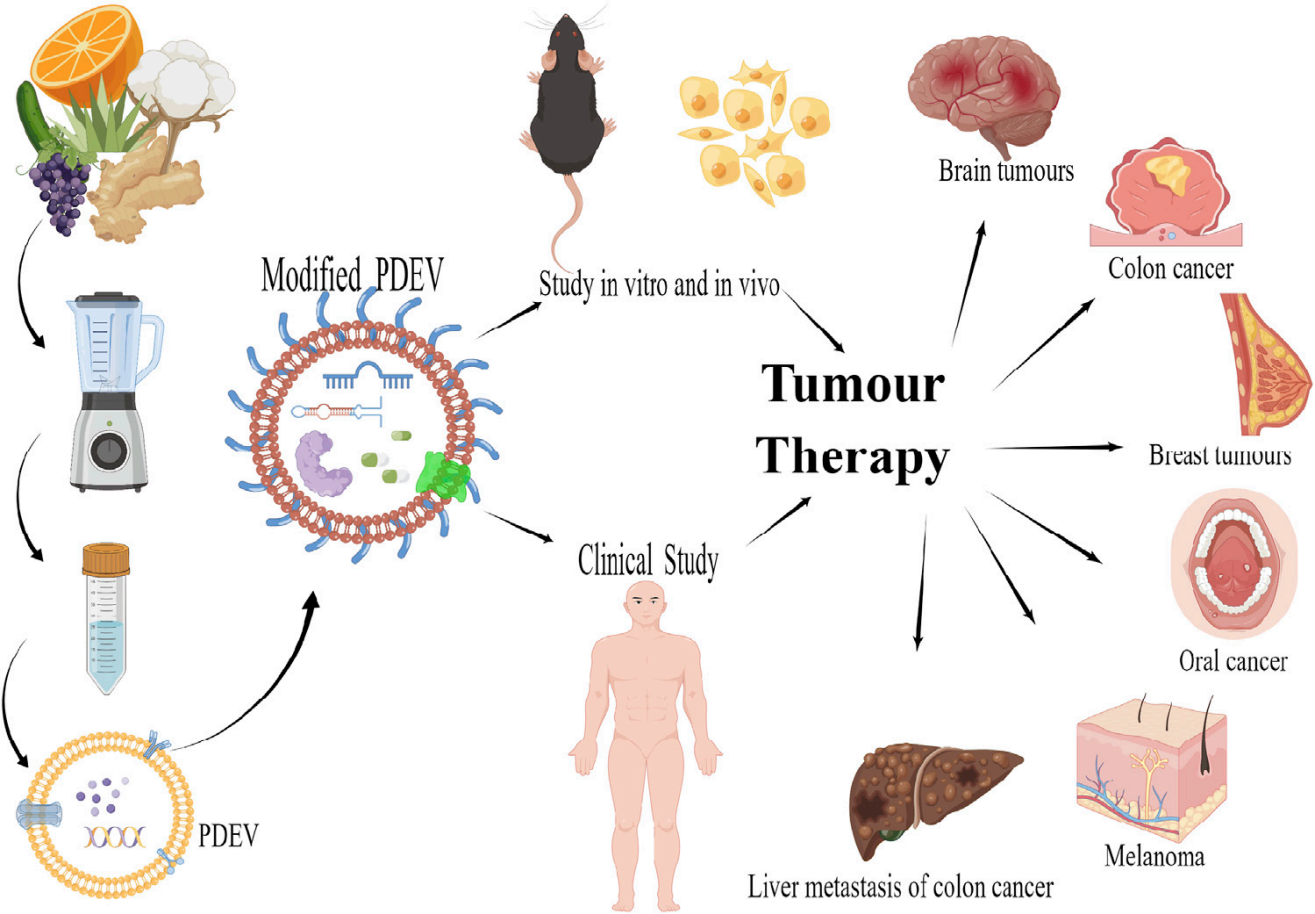
PDEVs as nano-delivery systems for tumour therapy created by figdraw.

The drug delivery system of PDEVs is still under development. Various cargoes such as nucleic acids, proteins and chemotherapeutic agents have been loaded into PDEVs and tested *in vitro* and *in vivo* in animal anti-tumour models as well as in the clinical treatment of cancer (Figure 1).

However, great challenges remaining in the study of PDEVs, and the application of PDEVs as drug delivery systems is still limited by technology. Firstly, there is a lack of uniform standard methods for the isolation and purification of plant-derived extracellular vesicles. Currently, ultracentrifugation, density gradient centrifugation, ultrafiltration, and particle size exclusion chromatography are commonly used for the extraction and purification of PDEVs in the laboratory. However, these methods are time-consuming and have low yields in the industrial conversion process. Secondly, the biological properties and transport mechanisms of plant-derived extracellular vesicles lack more comprehensive and in-depth studies. We have not yet identified the characteristic markers of PDEVs, while the surface proteins of PDEVs have not been studied in sufficient depth, which have led to a lack of clarity on the mechanism of action of PDEVs. A comprehensive understanding of the composition of PDEVs and consideration of a complete database would help to elucidate the biogenesis and mechanisms of PDEVs and provide reliable data to support the subsequent application of PDEVs. Furthermore, most studies have been based on cellular and mouse models, leaving unanswered questions about their long-term effects and physiological consequences. Extensive pre-clinical and large-scale clinical studies are therefore needed to ensure the safety of large-scale production use. Researchers also need to continue to investigate further in terms of improving drug loading efficiency, clarifying storage conditions, and increasing circulation time *in vivo*.

Note: Because of the lack of information about the biological origin, scientists have not yet unified the naming of PDEVs, and different terms are used in different literature to describe PDEVs such as nanoparticles (Zhang et al., 2016b), exosomes-like nanovesicles (Chen et al., 2022) and microvesicles (Alfieri et al., 2021). Several researchers (Pinedo et al., 2021) have called for the establishment of a standardised scheme to name plant extracellular vesicles, which has not been widely recognised. For the convenience of writing and understanding, in this mini-review, we will use the term ‘PDEV’ to refer to different names in the literature.
